# Comparing Two Novel LiDAR‐Based Indices for Quantifying Forest Structural Complexity

**DOI:** 10.1002/ece3.73605

**Published:** 2026-05-05

**Authors:** Tillman Reuter, Sebastian Seidel, Dominik Seidel

**Affiliations:** ^1^ Department for Spatial Structures and Digitization of Forests, Faculty of Forest Science and Forest Ecology University of Göttingen Göttingen Germany; ^2^ 44.moles GmbH Göttingen Germany

**Keywords:** box dimension, canopy entropy, comparison, fractal dimension, Hausdorff dimension, index

## Abstract

Forest structural complexity is critical for ecosystem functions, yet standardized metrics for its quantification remain elusive. This study compares two LiDAR‐derived three‐dimensional indices, the box dimension ($D_{b}$) as a fractal‐based measure, and canopy entropy (CE), an entropy‐based metric, to evaluate their methodological, computational, and conceptual differences. Using mobile LiDAR scans from 15m×15m forest plots in Maine, USA, and Nova Scotia and New Brunswick, Canada, we analyzed 170 point clouds to assess correlation, computation time, and theoretical underpinnings. Statistical analysis revealed a strong linear relationship between $D_{b}$ and CE (Pearson's r=0.823,p<0.001), with Deming regression indicating $\hat{\mathrm{CE}}=4.75\times D_{b}-1.07$. Also, CE computation averaged 40 times slower than $D_{b}$, scaling roughly linearly with point cloud size. Conceptually, $D_{b}$ reflects fractal dimensionality linked to physiological process optimization, while CE quantifies biomass distribution homogeneity. ${\mathrm{CE}}^{’}$s unit dependence on plot size limits cross‐study comparability, whereas ${D_{b}}^{’}$s dimensionless fractal interpretation offers broader intuitiveness. Both indices address sampling density bias but differ in parameterization and data efficiency. Despite ${\mathrm{CE}}^{’}$s theoretical novelty, it does not surpass $D_{b}$ in interpretability, precision, or speed, and its proposed advantage in capturing higher complexity remains unsubstantiated. Despite their conceptual distinctions, their strong correlation suggests competitive rather than complementary roles. Future research should explore biome‐specific variability and physiological links to ecosystem functions to refine their utility in forest management under climate change.

## Graphical abstract

This study compares two LiDAR‐derived forest structural complexity indices: the fractal‐based box dimension ($D_{b}$) and the entropy‐based canopy entropy (CE). Analysis of 170 plots revealed a strong linear correlation (*r* = 0.823) between $D_{b}$ and CE, but computation was much slower. While CE measures biomass distribution homogeneity, $D_{b}$ reflects fractal dimensionality already linked to physiological processes; $D_{b}$'s dimensionless nature offers broader interpretability, and did not demonstrate superior performance.

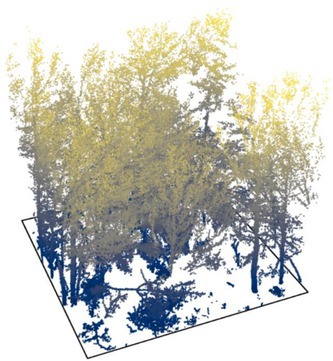

## 1 Introduction

Climate change is altering forest ecosystems around the world (Allen et al. 2010; IPCC 2019; Masson‐Delmotte et al. 2021). Direct effects like temperature rise, longer drought periods, more irregular precipitation patterns, and unforeseen floods (Masson‐Delmotte et al. 2021) damage forests more frequently (IPCC 2019). At the same time, people expect an increasing number of services from forests, such as timber production, carbon sequestration, atmospheric cooling, and biodiversity (IPCC 2019; Puettmann 2011). As forests are complex systems, all these functions are interrelated.

Forest structure is an ecosystem feature that was shown to correlate with primary production (Gough et al. 2019; Hardiman et al. 2011, 2013; Ishii et al. 2004; Siddiqui et al. 2025), biodiversity (Aponte et al. 2020; Atkins et al. 2018; Ishii et al. 2004; Lindenmayer et al. 2000; Lunow et al. 2026), ecosystem resilience and adaptability (McElhinny et al. 2005; Messier et al. 2013; Neill and Puettmann 2013; O'Hara and Ramage 2013; Seidel and Ammer 2023), stability (Musavi et al. 2017), and habitat suitability (Hinsley et al. 2009; Lee et al. 2023; MacArthur and MacArthur 1961). This puts forest structure in a central role for our understanding of forest ecosystems and their dynamics.

At the same time, the quantification of forest structure in a comprehensive way is not standardized. A large number of metrics is used and the metrics used have changed drastically over time, spanning from indices that can be obtained with just a measuring tape to indices that are based on advanced technology like Light Detection and Ranging (LiDAR), processing millions of data points (Seidel et al. 2011). Most forest indices, especially traditional ones, view forest structure either horizontally or vertically (Pommerening 2002; Pretzsch 2009). Due to the well‐established need for quantifiability of forest/canopy structure, many new indices have been developed (Richardson et al. 2014; Batchelor et al. 2023; Siddiqui et al. 2025), all with different use cases and objectives. The two forest metrics, that are chosen for this comparison, have placed their focus on evaluating forest structure holistically, equally in all three spatial dimensions while being suited towards Mobile Laser Scanning (MLS) LiDAR, namely the box‐dimension ($D_{b}$) and the canopy entropy (CE), introduced by Seidel (2018) and Liu et al. (2022), respectively. Both indices can be calculated based on laser point clouds obtained using one of the LiDAR platforms: mobile, handheld, backpack, unoccupied airborne or airborne laser scanning. Hence, they both rely on fused together point clouds in contrast to single‐perspective scans (terrestrial laser scanning), for which other structural metrics exist (Batchelor et al. 2023; Richardson et al. 2014; Seidel et al. 2020).

A recent study emphasizes the need for a unified methodology to assess forest structure in order to gain knowledge about the interrelations of forest structure with ecosystem functions (Ali 2019). In research, a method becomes established through use, thereby conferring a competitive advantage that new methods or technologies must overcome. Niche theory, developed in the field of ecology, has been applied to such dynamics (Geels 2002; Hannan and Freeman 1977; Kuhn 1994; Podolny and Stuart 1995; Tushman and Anderson 1986). Within this framework, the competition effect ensures that established methods further consolidate their position in the absence of disruption (Tushman and Anderson 1986). Consequently, for a newly introduced method to compete successfully, its disruption must therefore be large enough to outweigh the competitive advantage of the incumbents (Geels 2002).

As CE was introduced more recently than $D_{b}$, the method, which CE is supposed to compete against (Liu et al. 2022), it has to be so disruptive in order to become established within the landscape of forest structure metrics. To have a lasting impact, CE has to either be more convenient to use than already established indices, easier to interpret, have a wider range of applicability, have a higher resolution (be able to detect finer changes), or be cheaper in application. Overall, it has to be of minimum cost while of maximum outcome. It has to establish itself either alongside other indices or as a replacement for them.

In order to evaluate the performance of CE as a measure of holistic forest complexity, the following study examines these five facets by comparing it against the box‐dimension, an index already widely applied for the quantification of tree and stand structural complexity in recent years (Arseniou et al. 2021; Dorji et al. 2021; Heidenreich and Seidel 2022; Höwler et al. 2024; Mathes et al. 2023; Neudam et al. 2022; Saarinen et al. 2021; Seidel and Böttger 2023; Wildermuth et al. 2024). We structured this comparison into topics that were then answered using quantitative and qualitative methods side‐by‐side.

## 2 Material and Methods

### 2.1 Box Dimension

The box dimension, hereafter $D_{b}$, was introduced as an index of mathematical complexity of trees and forests using LiDAR point clouds (Seidel 2018; Seidel, Ehbrecht, et al. 2019; Guzmán et al. 2020), relying on the work of the mathematician Benoît Mandelbrot (Mandelbrot 1983). First used as an index of single tree structural complexity based on ground‐based LiDAR data, it was consecutively applied to tree groups (Seidel, Ehbrecht, et al. 2019) and entire forest stands (Heidenreich and Seidel 2022; Neudam et al. 2022; Stiers et al. 2020), and expanded to airborne LiDAR data (Camarretta et al. 2021; Seidel et al. 2020).

In mathematics also known as Hausdorff‐Dimension, Fractal Dimension or Box‐Counting Dimension, the metric $D_{b}$ aims at describing the number of dimensions a structure aims to uniformly fill. However, when applied to three‐dimensional (3D) objects, as it is a continuous measure, it rather estimates the uniformity of the filling of the three dimensions (Mandelbrot 1983). In the case of laser point clouds, which only contain points on the surface of objects, as that is what can reflect the laser beams, the index considers the filling of 3D space by the surface of the forest vegetation. In its very essence, $D_{b}$ is therefore a holistic measure of forest structural complexity.

**FIGURE 1:**
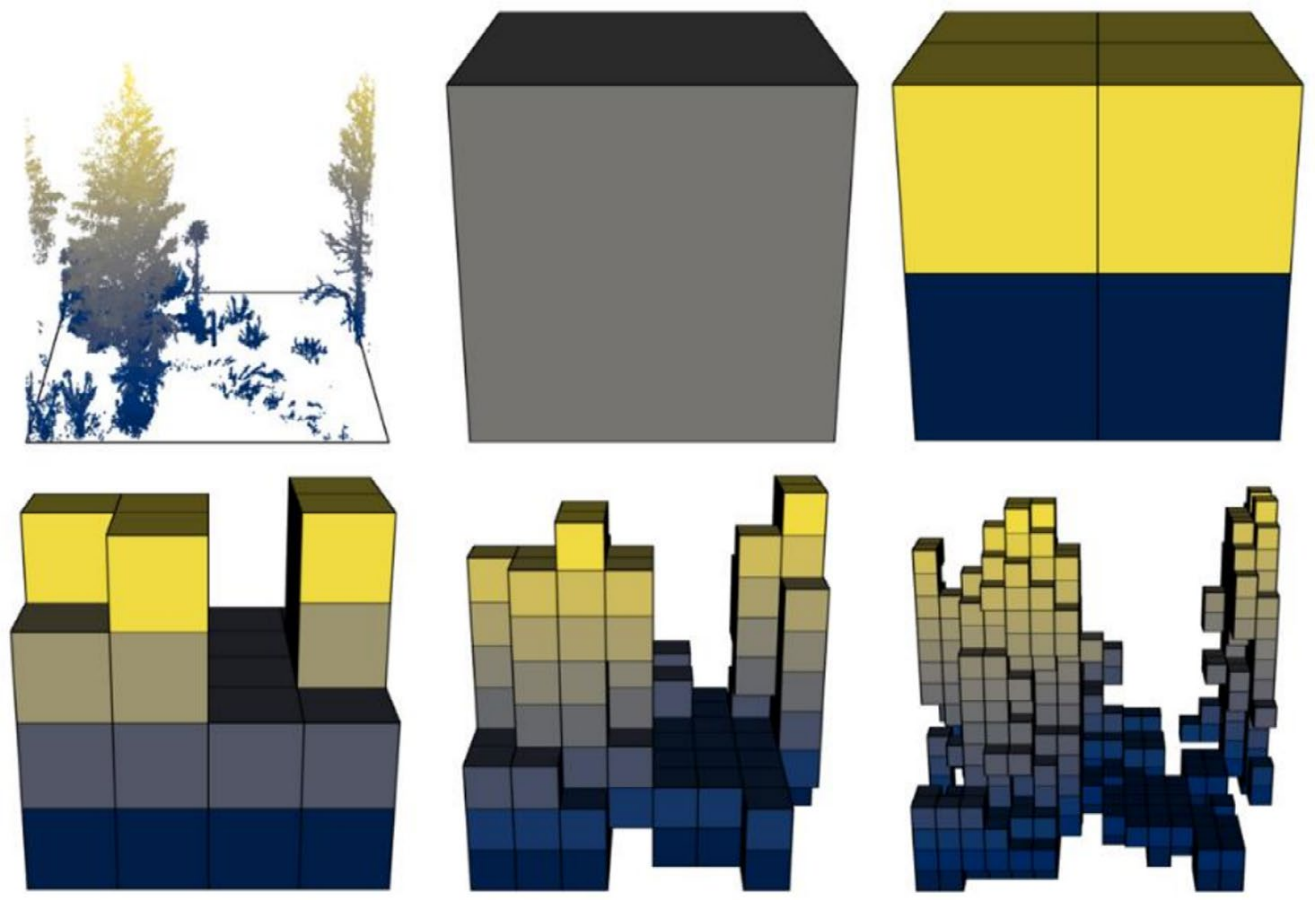
Process of dividing the point cloud into voxels (boxes) of decreasing size, displayed are the occupied voxels $\left(N\right)$ for decreasing voxel sizes from upper left in a row‐wise manner to the lower right.

**FIGURE 2:**
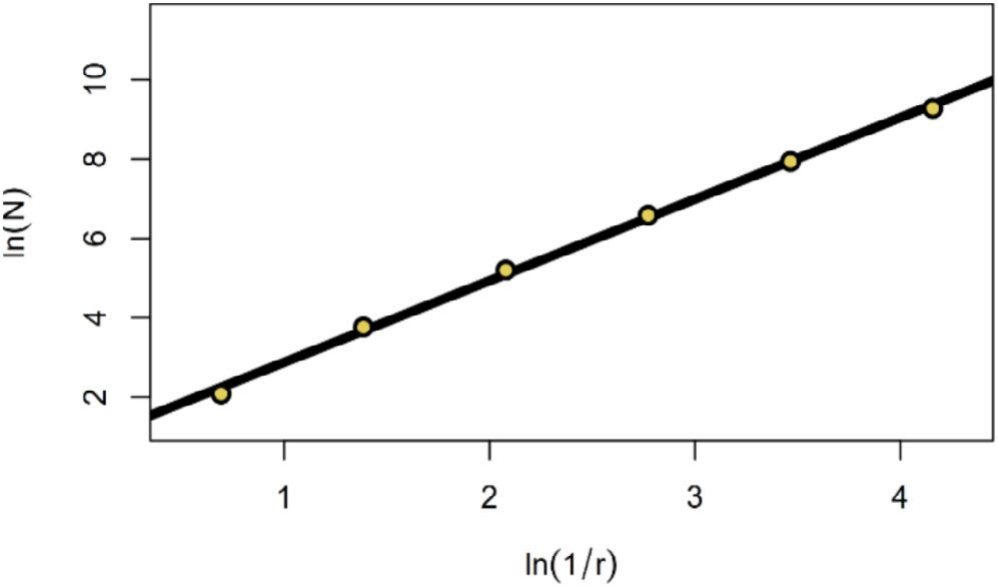
Example regression of $\ln\left(N\right)$ against $\ln\left(1/r\right)$. The slope of the regression line represents the box‐dimension ($D_{b}$) of the point cloud of interest.

$D_{b}$ is computed by applying a 3D grid to the point cloud. In series, one now divides the grid sequentially into voxels (3D pixels) of decreasing size, with their side‐length starting at the point cloud extent and then always decreasing by factor 0.5 (see Figure 1). At every step, the number of occupied voxels (voxels containing at least one point) is obtained. Then, using the two vectors side‐length (r) and number of filled voxels (N), a linear model with r inversed and then both variables log‐transformed is calculated (see Figure 2). The slope‐coefficient $\beta_{1}$ of the resulting regression formula (Equation 1) is the $D_{b}$.

(1) $$\ln\left(N\right)=\beta_{0}+\beta_{1}*\ln\frac{1}{r}+\epsilon_{i}$$

To phrase this differently, $D_{b}$ is the exponent, by which *N* grows with regard to $\frac{1}{r}$, or $D_{b}$ is the ratio of the exponents of N and $\frac{1}{r}$ while decreasing r (see Equation 2).

(2) $$D_{b}=\lim_{r\to 0}\frac{\ln\left(N\right)}{\ln\left(\frac{1}{r}\right)}$$

### 2.2 Canopy Entropy

Liu et al. (2022) introduced CE as an entropy‐based forest canopy structural complexity index. It works by estimating the Probability Density Function (PDF) of the distribution of biomass along the three orthogonal planes that make up the three‐dimensional point cloud (xy,xz,yz) using Kernel Density Estimation (KDE). By multiplying these two‐dimensional PDFs with their natural logarithm, they are distorted towards rewarding more homogenous PDFs with a bigger double integral (volume under the surface, see Equation 3). These three double integrals of xy, xz, and yz are then combined using the pythagorean theorem into one holistic CE value representing all three dimensions (see Equation 4).

(3) $${\mathrm{CE}}_{\mathrm{xy}}=-\int \int p\left(x,y\right)\times \ln\left(p\left(x,y\right)\right)\text{dxdy}$$

↓

(4) $$\mathrm{CE}=\sqrt{CE_{\mathrm{xy}}^{2}+CE_{\mathrm{xz}}^{2}+CE_{\mathrm{yz}}^{2}}$$

Before this PDF estimation, the point cloud goes through a resampling procedure. Because not all objects of a forest stand are at the same distance to the LiDAR measuring device, the objects of the forest canopy are hit by a varying flux density of laser rays. This means closer objects are represented by more points in the point cloud than more distant ones. From now on, this effect is called Sampling Density Bias (SDB). Because this effect would show in the PDF estimation, it is accounted for via resampling of the points based on the distance of the points to one another. Because the SDB mainly occurs along the z‐direction, it is only accounted for in this direction. The test used in Liu et al. (2022) is based on the Mann–Kendall‐Test (MK‐Test) and was adapted to detect linear trends in time‐series‐analysis (Hamed and Ramachandra Rao 1998).

The point cloud is separated into horizontal layers of 1m thickness and the average point distance (to the nearest neighboring point) is calculated for each layer. If the MK‐Test detects a linear trend with p<0.05, the point cloud is declared biased. In this case it is voxelized (like in Figure 1) with a voxel size of the biggest average point distance of all layers. Instead of the original point cloud, the center points of the voxels are then considered in the PDF estimation. The MK‐Test and subsequent voxelization is repeated with voxel‐size always increasing by 10 until the MK‐Test no longer detects a linear trend (p<0.05) (Liu et al. 2022).

### 2.3 Study Area and Data

**FIGURE 3:**
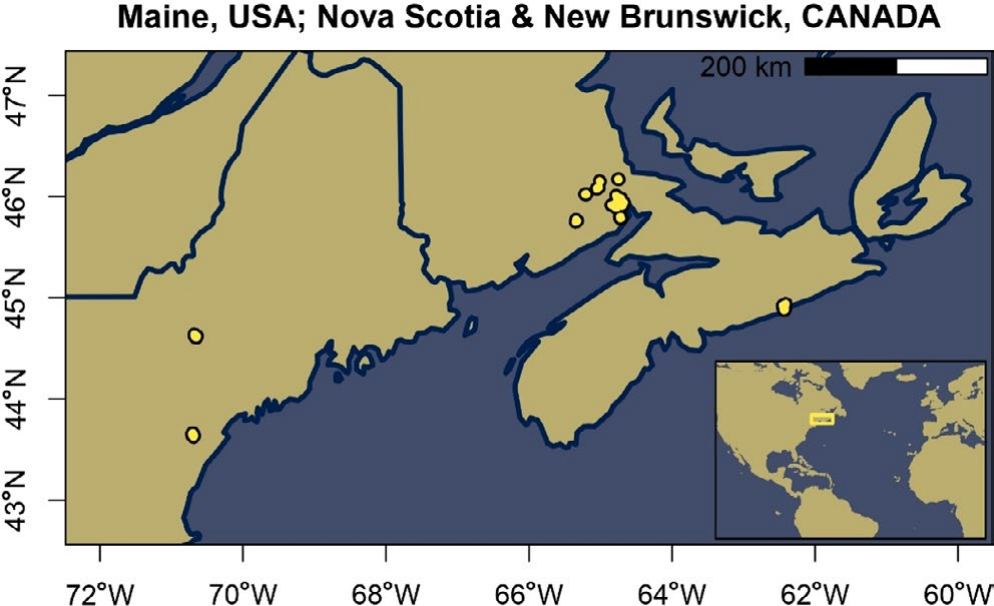
Map of the spatial distribution of the forest sites along the north‐east coast of North America.

To compare both indices, point clouds were obtained in forests spread over the north‐east coast of North America, specifically Maine, USA, and Nova Scotia and New Brunswick, Canada (Figure 3).

The forests therefore represent the biomes “mixed and temperate forests” (Lapola et al. 2008; Matthews 1983; Ramankutty and Foley 1999) in “Dfb climate” (snow climate, fully humid, hot summer) following the Köppen‐Geiger classification (Kottek et al. 2006). The Nova Scotia sites are conifer‐dominated Acadian forests with around 30% hardwood, influenced by storm events and heavy use, which results in below average vegetation density. The areas in New Brunswick are distinct mixed deciduous forests, are older, and have a higher stock than those in Nova Scotia. The forests in Maine are also Acadian forests, mostly deciduous with conifers in small groups. The scanned plots were strategically placed to represent their surrounding forest ecosystems.

**FIGURE 4:**
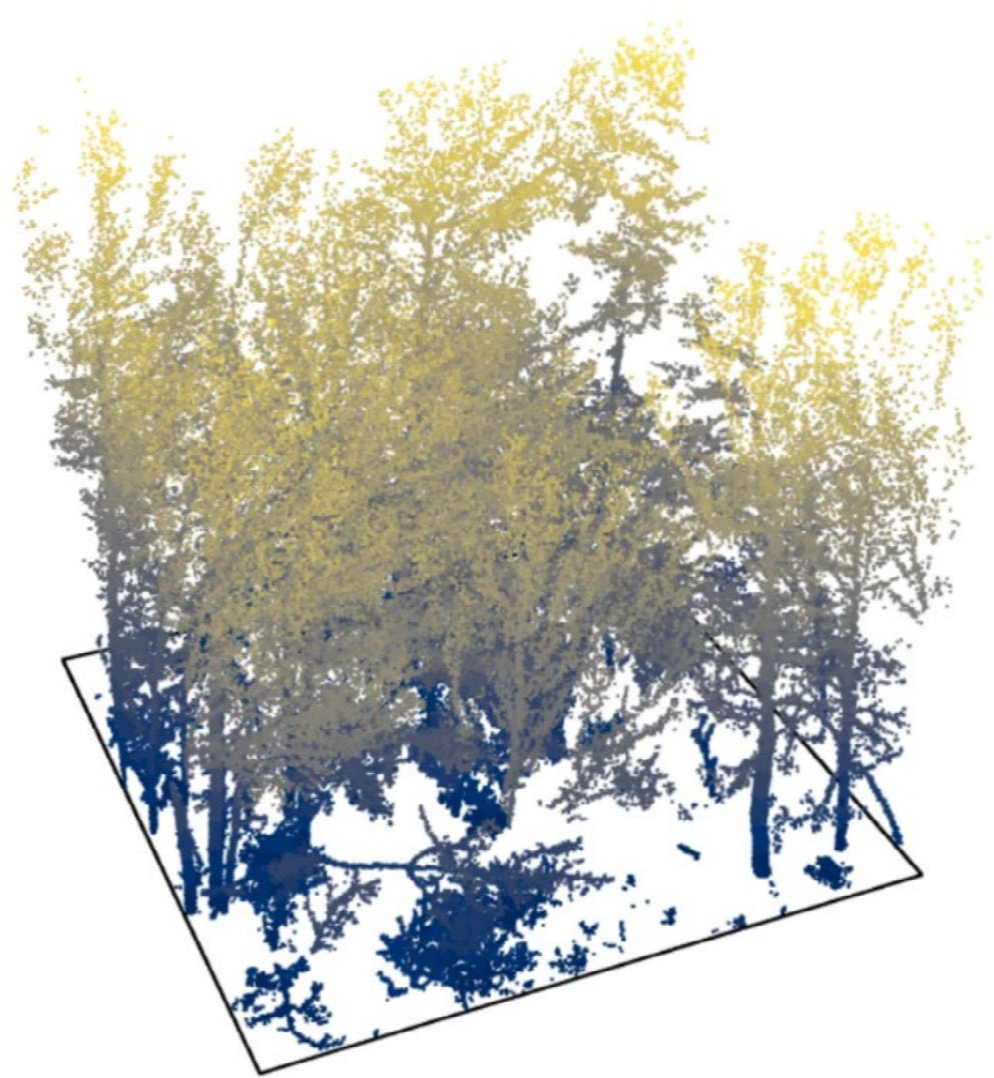
Example MLS point cloud, cut to a 15 × 15 m vertical column around the center point. The color gradient visualizes the height above ground. The point cloud is normalized and ground points are removed.

On all plots a point cloud of 15m×15m in horizontal extent was obtained using a handheld Geoslam ZEB Horizon MLS device (FARO Technologies Inc., Lake Mary, USA 2026). Starting in one corner of the 15m×15m plot, the plot was first surrounded and then traversed following the standard scheme as described in Neudam et al. (2022) in order to capture the forest stands entirely and uniformly. The height of the point clouds varies and is as high as the canopy top of the regarding forest plot. The resulting point clouds were converted into .laz‐files using the Geoslam Hub software (FARO Technologies Inc. 2025) for further processing. An example point cloud can be viewed in Figure 4.

### 2.4 Data Preparation and Index Computation

Prior to computing the indices, the point clouds were normalized using the LiDAR360 software on default parameters for hilly terrain (GreenValley International Inc. 2022) and the classified ground points were removed.

The computer code for the index computation consists of provided python code by Liu et al. (2022) and Seidel (2018) and own “python” and “R” code, which wraps around the algorithms and hereby accounts for computation time (Python‐Software‐Foundation 2025; R‐Core‐Team 2018). Different to what was described in Seidel (2018) but in accordance with the code presented in Arseniou et al. (2021), the first point (representing the leftmost step in Figure 1, $\left(0,x\right)$ in log/log‐form) was not considered in the regression analysis of the $D_{b}$‐algorithm.

### 2.5 Quantitative Comparison

The quantitative comparison can be split into a statistical mapping of the indices against each other and a computation time analysis.

#### 2.5.1 Statistical Comparison

The aim of the statistical analysis was to capture the relationship of the two indices and also to quantify how much they are alike by value, how much they can be explained by one another. Many statistical regression methods demand the declaration of a dependent and an independent variable, which creates hierarchy between the variables. This hierarchy is significant because regression results vary if the order of variable declaration is changed (regression towards the mean). To be immune to this bias, caution was taken to avoid using hierarchical statistical methods in the analysis presented here.

There are regression methods that don't depend on this variable hierarchy and hence fit relationships symmetrically. Among those are Total Least Squares regression and (rank) correlation coefficients. The former was developed as a bivariate statistical test, that models errors in both x‐ and y‐direction. This is achieved by constructing the residuals orthogonally to the regression line instead of vertically, as Ordinary Least Squares regression does. A consequence of this different geometry is that the order of x and y can be switched without the model fit behaving differently with regard to the point cloud. Because the calculated regression line can be described by a bijective function if not completely flat, it can be parameterized both as $f\left(x\right)=y$ and as $f\left(y\right)=x$. Deming Regression extends this method by accounting for different error variances in x and y through their error variance ratio. This ratio, which needs to be specified, affects the angle at which the regression line meets the residuals (Linnet 1993). To ensure an unbiased comparison, it is assumed that the index's measurement error is proportional to the total range of respective index values in the forest stand data, i.e., both methods have the same signal‐to‐noise ratio. Therefore, the error ratio is assumed to be equal to the range ratio of CE and $D_{b}$. This calculates to:

(5) $$\delta =\frac{\max\left(\mathrm{CE}\right)-\min\left(\mathrm{CE}\right)}{\max\left(D_{b}\right)-\min\left(D_{b}\right)}=\frac{8.12}{1.99}=0.24$$

Because it allows for a regression line, Deming‐Regression has higher interpretability than (rank) correlation coefficients like Pearson's correlation coefficient, Spearman's Rank correlation coefficient and Kendall's τ, but is limited by relying on a linear relationship. In contrast, correlation coefficients deliver a metric of goodness‐of‐fit.

Because forest stands are spatial objects and ecosystem features tend to spatially auto‐correlate, the Independence Assumption of Statistical Modeling is violated. Also, the forest scans span over several forest sites, so they are not equally or even similarly geographically distant to one another. To account for this violation, a control model was calculated where the forest site was introduced as a covariate. This way, for each of the four spatially distinguishable forest sites, Deming‐Regression models were calculated, and the regression lines were evaluated.

Therefore, the first step of the statistical analysis was to check for linearity in the data. Visual evaluation of the scatter point cloud of CE and $D_{b}$ was used to achieve this. After approving the linear relationship, Deming‐Regression was the preferred method for analysis, because it allows the translation of index values into one another. In parallel, Pearson's correlation coefficient was used to evaluate goodness of fit.

In the case of a non‐linear relationship Spearman's Rank correlation coefficient and Kendall's τ would have been used to make sense of the correlation by evaluating monotonicity between the indices.

The R programming language was used for all statistical analysis (R‐Core‐Team 2018) along with the software packages “viridis” and “mcr” (Garnier et al. 2024; Potapov et al. 2024).

#### 2.5.2 Computation Time

Computation time was measured for the calculation of both indices for all 170 point clouds. Table 1 describes the specifications of the machine that was used for the index computation. To not only answer the question, which index calculation takes longer on average but also what a potential difference is caused by, we also investigated how both algorithms scale with point cloud size. To achieve this, standard linear models as well as non‐linear models (Ordinary Least Squares) solving for the exponent of N, were calculated for both indices:

(6) $$\hat{t_{\mathrm{CE}}}=aN^{b}$$

(7) $$\hat{t_{D_{b}}}=aN^{b}$$

**TABLE 1:** Machine specifications.

Software	Version
Python	3.10.11
laspy	2.5.4
matplotlib	3.10.0
numpy	2.2.0
open3d	0.18.0
pandas	2.2.3
pymannkendall	1.4.3
sklearn	1.6.0
R	4.4.3
reticulate	1.39.0
VSCode	1.97.2
RStudio	2024.12.0
OS	Windows 11
**Hardware**	**Specifications**
CPU	11th Gen Intel Core i5‐1135G7@2.40GHz 1 of 4 cores
RAM	16.0GB

The choice of model is based on the assumptions that N influences the computing time exponentially, i.e., linearly, quadratically or cubically. It is also assumed that $\lim_{N\to 0}t=0$; therefore, the regression intercept is 0.

### 2.6 Conceptual Comparison

To examine the conceptual differences and similarities, both indices were also explored conceptually. The following aspects are investigated: the algorithmic complexity, the efficiency of data use, the interpretability, the units and the physiological interpretation. It was also assessed in a qualitative way how the methods handle the Sampling Density Bias. Sampling Density Bias (SDB) is a significant hurdle when processing point clouds. The fact that the intensity at which objects are captured by the LiDAR device varies across the point cloud complicates the process of structure quantification.

Also, both indices depend on agreed‐upon parameters. Because parameterizing a function adds complexity to the method which goes against simplicity and therefore interpretability, the number of parameters of each index was also discussed.

## 3 Results

This results section is written in the language of evidence (Muff et al. 2022).

### 3.1 Relationship Between $D_{b}$ and CE

**FIGURE 5:**
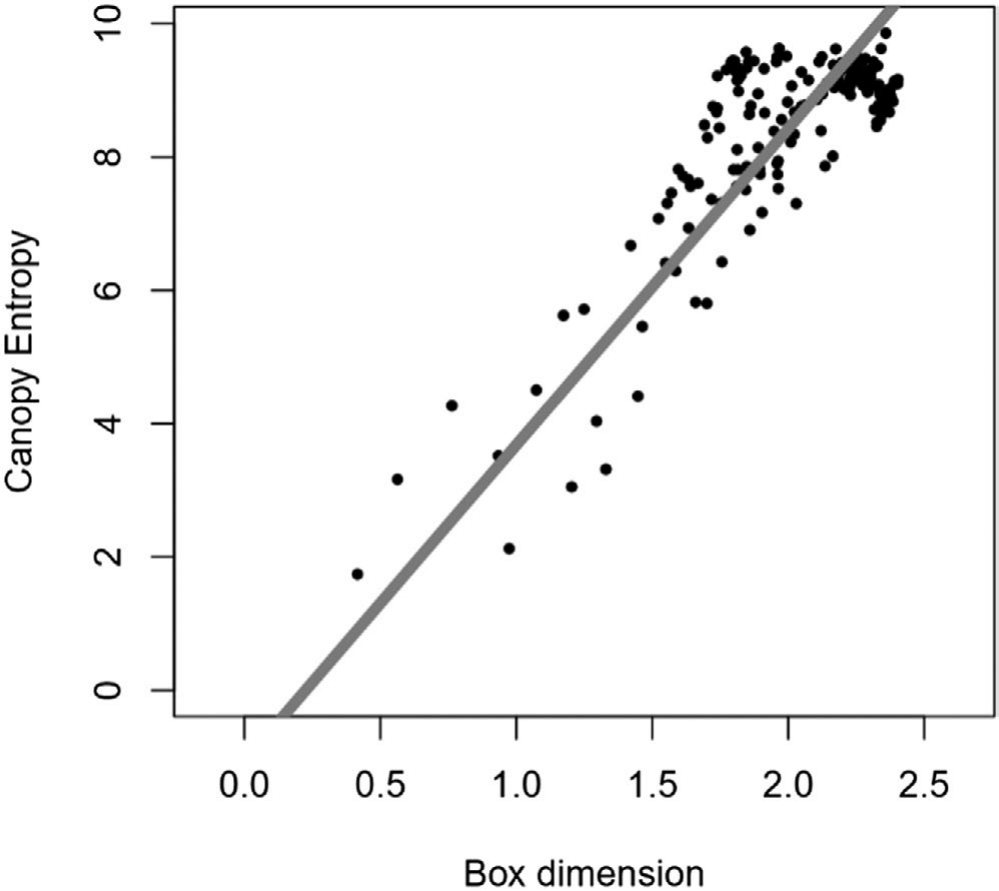
Scatter plot of canopy entropy over box dimension with Deming‐Regression line highlighted in gray.

The scatterplot in Figure 5 approves the linear relationship between CE and $D_{b}$, which is needed to use Pearson's correlation coefficient and Deming regression.

Pearson's correlation coefficient calculates to r=0.823 ($R^{2}=0.677$) and is very strongly evident (p<0.001). The Deming‐Regression's results can be viewed in Table 2 and its regression line in Figure 5. Deming‐Regression does not permit the formulation of statements regarding *p*‐values due to its lack of a Null Hypothesis. However, this is of negligible concern, as the correlation's evidence has already been substantiated. The parameter estimates serve as translation between the indices in both directions. The regression formulae therefore constitute as:

(8) $$\hat{CE}=4.75D_{b}-1.07$$

(9) $$\hat{D_{b}}=0.211\mathrm{CE}+0.225$$

**TABLE 2:** Results of the Deming regression, containing the parameter estimate (EST), its standard error (SE) and lower (LCI) and upper (UCI) boundary of a 95%‐confidence interval.

PAR	EST	SE	LCI	UCI
Intercept	−1.07	0.51	−2.1	0.0
Slope	4.75	0.25	4.2	5.2

**FIGURE 6:**
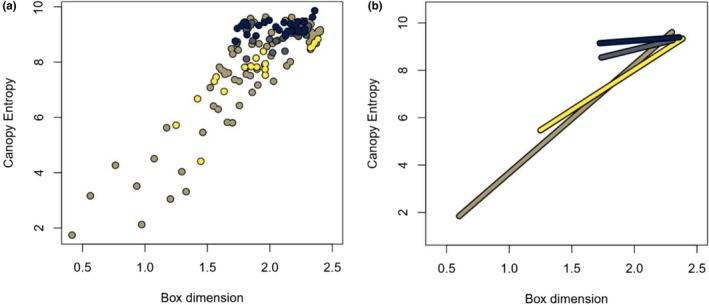
(a) Scatterplot of CE and $D_{b}$, color representing the four different forest sites. (b) Regression lines (Deming) of the four forest sites.

The mixed model, to validate that this linear trend is not just a statistical artifact of the violation of independence and identical distribution, showed comparable results which can be seen in Figure 6. The forest stands of generally higher structural metrics proved to have less steep regression lines in the here chosen plotting perspective.

### 3.2 Computation Time

**FIGURE 7:**
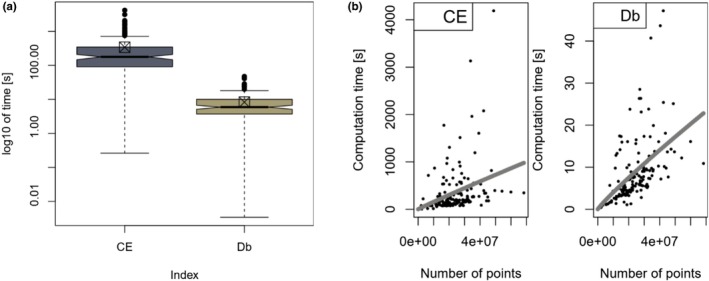
(a) Boxplot of computation times of CE and $D_{b}$ $\left[s\right]$, $\mathrm{lo}g_{10}$‐transformed, box‐crosses represent the distribution mean, notches represent the medians' 95‐CI. (b) Computation times of CE and $D_{b}$ against the number of points N.

Table 3 shows the time it took for the algorithms to read in the point clouds and calculate the indices. The index calculation took on average 40‐times longer for CE than for $D_{b}$. When including the time of loading the point cloud into python or R objects, this factor reduces to 15. The distributions of computation times per index can also be viewed in Figure 7a.

**TABLE 3:** Computation time per cloud $\left[s\right]$, average and interquartile range in brackets.

Procedure	$D_{b}$	CE
Reading point cloud	$15.1\left[7.60;17.9\right]$	
Index calculation	$8.3\left[3.73;9.99\right]$	$334\left[87.5;340\right]$
Sum	$24.6\left[12.6;31.2\right]$	$356\left[100;370\right]$

When evaluating computation times with regard to the number of points per scan, it shows that both indices' algorithms scale roughly linearly (see Figure 7b). Specifically, the estimated exponents of N (0.96 and 0.91) are close to 1 (which would indicate a perfectly linear relationship) and also their standard errors (0.25 and 0.13) make their possible ranges overlap largely.

Although the regression lines do not seem to capture the underlying trend well, out of all bi‐parametric model configurations, the presented models explained the most variance (see Table 4). With 68% against 38% of explained variance, cloud size plays a much bigger role in the computation time of $D_{b}$ than of CE.

**TABLE 4:** Results of the non‐linear regression (Equations 6 and 7) for the relationship of computation times against *N*.

Index	PAR	EST	SE	*p*	$R^{2}$
CE	a	3.2e−5	3.2e−6	<2e−16	0.38
	b	0.96	0.25	2e−4	
$D_{b}$	a	1.8e−6	1.0e−8	<2e−16	0.68
	b	0.91	0.13	2.69e−10	

### 3.3 Dealing With Sampling Bias

Both indices have their way of accounting for SDB. While CE resamples the point cloud with a “spatial thinning” approach until no linear point distance trend in z‐direction is detectable, $D_{b}$ uses a minimal voxel size, which is, as could be shown by Mathes et al. (2023), a means to limit occlusion effects and other distance related effects.

Mathematically, the CE‐algorithm would also work by fixing the spatial thinning to 0.5 and circumvent the MK‐test, or vice versa, the $D_{b}$‐algorithm could also apply the MK‐test from the CE‐algorithm and use the resulting voxel size as minimum voxel size. Therefore, the approaches to account for SDB are not inherently linked to the respective algorithms' nature, but interchangeable. Because ${\mathrm{CE}}^{’}$s algorithm aims to model the point distribution based on exact point locations, it depends on the data in its initial biased form. Consequently, the algorithm is obliged to modify the data, which comes with pitfalls.

First, the statistical power of the MK‐based test, which is used for the resampling process of CE, is affected by sample size. A smaller sample‐size, in the case of this index the number of 1m‐layers, decreases the possibility of the test to detect SDB. Unfortunately, a non‐evident *p*‐value (p≥0.05) doesn't allow for the rejection of the $H_{1}$‐Hypothesis. In the case of the MK‐test: To conclude that p≥0.05 indicates that there is no linear trend is statistically incorrect. Null‐Hypothesis‐Significance‐Testing (NHST) only allows for the rejection of $H_{0}$, if p<0.05.

Secondly, the CE algorithm only measures Sampling Density Bias in the z‐direction (height), while it can actually occur in all three spatial dimensions. With LiDAR methods, where the device travels horizontally while scanning and therefore covers the xy‐plane sufficiently uniformly (mobile laser scanning, backpack laser scanning, airborne laser scanning), SDB hardly occurs in x‐ or in y‐direction. Nevertheless, because the distance of the scanner towards objects on the outskirts of the scan is on average bigger in x‐ and y‐direction than in z‐direction, the SDB detection method of CE does not quantify SDB completely. In consequence, the resampling via voxeling is performed too fine, and canopy objects in the horizontal corners are underrepresented in the PDF‐estimation. It is to be kept in mind that this limitation of CE's approach of accounting for SDB only has meaningful effect in scans derived from horizontally stationary LiDAR‐devices (single scan TLS, stitched TLS), because horizontally moving LiDAR‐devices (MLS, UAVLS, ALS) show far less horizontal SDB.

By accounting for SDB, both index algorithms reduce the usable data of the point cloud. While $D_{b}$ fixes the minimum voxel size to a side length that ensures the bias to be excluded, CE aims to be more flexible by considering the bias just as much, so that it is sufficiently accounted for. Ultimately, this gives CE an advantage in data usage efficiency for point clouds with low Sampling Density Bias. Because this bias is created by the relative difference in distances of objects to the LiDAR device, Mobile Laser Scans with low canopy height and also Airborne Laser Scans from far above the canopy have such a low bias. Mathematically, higher data usage efficiency results in a higher index sensitivity to fine forest structure changes.

### 3.4 Physiological Interpretation

Physiologically, $D_{b}$ is linked to the interaction between the forest canopy and the atmosphere. Parallel light rays are most effectively captured by a plane facing the origin of the light rays, as this is the minimal size object to capture the most light. This plane, as all planes, calculates to a $D_{b}$ of 2. Processes like gas‐exchange are optimized by the plant interacting as much as possible with the atmosphere, filling the three‐dimensional space with surface, which results in a $D_{b}$ approaching 3. The most efficient balance between light capturing, self‐shading and gas exchange should result in a $D_{b}$ of close‐to but less‐than 2.72 (Seidel, Annighöfer, et al. 2019). This directly makes $D_{b}$ interpretable. Because ecosystems optimize their surface complexity towards this value, any deviation can be interpreted as disturbance, e.g., by humans.

Because CE actually measures homogeneity in the distribution of matter in three‐dimensional space instead of mathematical complexity, the causal chain of interpretation slightly differs. On the one hand, an organism that maximizes fractal dimension also maximizes uniformity of its space‐filling, and an organism of zero mathematical complexity also has zero homogeneity. While at the edges, the interpretations of both indices line up, it is in between that their inherent meaning is different. Because natural systems don't have the incentive to approach maximum homogeneity, a CE‐value does not relate to ecosystem functioning, like $D_{b}$ does.

### 3.5 Number of Parameters

As $D_{b}$ works with a simpler algorithm, it also has less need for parameterization than CE. To be exact, $D_{b}$ is parameterized for the minimum voxel size, here at 0.5m. This is a balance of sample size in the linear regression plot on the one hand and safeguarding from Sampling Density Bias and occlusion on the other hand.

CE parameterizes the layer width and the *p*‐value in the resampling process with 1m and 0.05 respectively. In the PDF‐Estimation, the bandwidth of the KDE‐algorithm is fixed to 0.2m. This value is the result of a sensitivity analysis of Liu et al. (2022).

### 3.6 Units

The unit of $D_{b}$ can be described as the float number of dimensions the forest is filling uniformly, like described in Hausdorff (1919) as Hausdorff dimension of mathematical topologies. This number of dimensions is quantified continuously, similar to measures like Shannon's effective species richness, which originated in capturing entropy in information‐theory (Bartsch and Röhrig 2015) or Effective Number of Layers, a forest metric for vertical layering (Ehbrecht et al. 2016). This way, an object that is effectively somewhere between a line and a plane can be quantified with 1<x<2. This interpretation also intuitively delivers boundaries to the metric. A point cloud of three dimensions (x,y,z) can approach no fewer than 0 and no more than 3 dimensions. Trees, because of their objective of maximizing surface to volume, have an upper limit of 2.7268 (Seidel, Annighöfer, et al. 2019), which is the Hausdorff dimension of the Menger Sponge (Carfì et al. 2013).

CE on the other hand, mathematically describes the Pythagorean combination of three volumes under the PDF surfaces, so ultimately it is a volume with the unit $m^{3}$ if m is the length unit of the point cloud.

Because PDFs have an area of 1 by definition, their values are indirectly proportional to the extent of the point cloud. The calculated value of CE therefore depends on the forest plot side length and on the total height. CE‐values of forest plots with different sizes can therefore not be compared intuitively. A larger cloud automatically returns a larger CE‐value.

### 3.7 Ability to Capture the Underlying Theory

What the box‐counting‐algorithm of $D_{b}$ aims to capture is fractality. Natural objects, like trees, can only be fractal to some extent (Shenker 1994), limited by the minimum size, their organs can have. This explains why the points of Figure 2 are not on a straight line. If forest stands were fully fractal, it would not matter if the slope between the second and the third, or the fourth and the fifth points would be taken as $D_{b}$. The linear regression ultimately averages the single slopes. As can be seen in Figures 1 and 2, during some steps, the number of filled voxels grows with power of 3, and during some steps with a smaller exponent.

There is another effect that applies to forest scans with much bigger side‐length than canopy height. Imagine a forest scan with side‐length 20m and canopy‐height 5m. The first two steps of voxeling (r=10,r=5) would approximate the laser point cloud towards a plane and only then would the three‐dimensional fractal nature of the canopy come into play. Because of the regression method, this dampens the $D_{b}$ of the forest scan. The consequence is that when increasing the side‐length further than the canopy height, $D_{b}$ decreases.

## 4 Discussion

### 4.1 Unexplained Variance

The residuals of the correlation plot (Figure 5) can be caused by first, deviation in the indices' ability in capturing their underlying theory, and second, in the differences between the underlying theories. How these effects come together cannot be evaluated through the methods of this study.

### 4.2 Mixed Effects Modeling

Although the mixed effects model (including forest site as a covariate, see Figure 6) showed different results as the reference model, the principal character of the regression is the same. For statistical reasons, the reference model is more powerful as it has less degrees of freedom. While the reference model quantizes $\frac{D_{b}}{\mathrm{CE}}$ with $\frac{1}{4.75}$, and this ratio represents all used data, it should be taken with caution, especially at the edges. Extrapolating into realms not investigated here is strongly discouraged. Also, as is described in Chapter 4.3, the ratio does not hold across possible experimental setups. What is rather the main result of the quantitative comparison is the amount of deviance that can be explained, which is shown by the Pearson's correlation coefficient. Here, a much stronger correlation appeared than in Liu et al. (2022), 0.68 versus 0.32. This could be explained by different properties of the different continents' forests (East Asia and Eastern North America), generally different ranges of forest structure put to the test, or the different regression approaches. Also, Liu et al. (2022) partly used simulated point clouds (9 of 30), which appear to have a different index relationship than the natural forest stands.

### 4.3 Scaling

The two indices are both affected by scan side‐length, yet for different reasons and with different consequences. While CE automatically rises with plot‐size because of the PDF‐Estimation, $D_{b}$ decreases with plot‐size (if plot‐size exceeds canopy height). This ultimately constrains the universality of the index‐ratio calculated in Chapter 3.1. It only holds for a plot size of 15 m. Solutions to overcome this bias could include scaling the point cloud to a unit‐length before processing (CE) or using non‐cubic voxels (e.g., rectangular cuboids) as voxelization‐units ($D_{b}$). These would be parameterized by the canopy‐height and preserve their allometry throughout voxeling.

That the effect of plot‐size on index‐values hinders cross‐study comparison already shows in this study's results. Liu et al. (2022) used 20m×20m‐scans and simulated clouds, calculated both indices and compared them. That their resulting relationship differs from this study can therefore be at least partially explained by scaling.

### 4.4 Index Range

CE was introduced partly because it is supposed to be able to capture forest structure, where $D_{b}$ reaches its upper limit (Liu et al. 2022). This hypothesis could theoretically be answered in a quantitative way by testing for a non‐linear relationship of the indices in a pairwise comparison. Although both Liu et al. (2022) and the authors of this study, to this moment, only applied linear regression (OLS and TLS), the question about this edge phenomenon of the correlation can nevertheless be partially answered. The mixed model, including forest site as a covariate, shows that the relationships between the indices have less steep slopes in forests of generally higher values (see Figure 6b), indicating that the relationship of CE and $D_{b}$ either depends on the structural variation between the stands of the different forests or that the relationship's shape actually tends to non‐linearity towards higher structural values. The analysis of Liu et al. (2022) indicates the same edge‐case behavior. If one looks only at their scanned forest stands and ignores the simulated ones, the relationship seems to curve towards a saturated CE while still increasing $D_{b}$. Both these findings contradict the hypothesis, that CE is able to capture higher forest structural complexity than $D_{b}$. However, until an in‐depth analysis of highly structurally complex forests has been performed, this debate is ongoing. Also, a changing slope of the relationship alone doesn't settle the argument. If with a decrease in slope comes a proportional decrease in statistical error, the indices' ability to map forest structure remains. Only a completely horizontal/vertical relationship yields saturation.

### 4.5 Computation Time

Computation time is important regarding applicability and practicability. The above calculated ratio of computation times between the two algorithms proves to be substantial and should be considered when choosing a method for forest canopy structure quantification. Nevertheless, it is important to mention that descendants of the $D_{b}$‐algorithm have been computationally solved for a much longer time than ${\mathrm{CE}}^{’}$s algorithm. It could be possible that box‐counting‐algorithms have gone through code‐optimization in a magnitude, which ${\mathrm{CE}}^{’}$s algorithm did not have the time for yet. Namely, the methods employed there (MK‐test, KDE) were not intended for this particular purpose and therefore might still yield potential to be optimized for quicker computation. Computation times are never steady over time, as software develops and hardware generally becomes cheaper and faster. The average computation times of this study (see Figure 7a) might right now limit the usage of CE but with further development of technology, the relevance of this difference might fade in the future.

The higher unexplained variance of the $t{\left(N\right)}_{\mathrm{CE}}$ model in comparison to the $t{\left(N\right)}_{D_{b}}$ model is probably caused by CE's resampling of the point cloud which takes variable time because of its iterative design and is independent from point count. The almost linear scaling of computation time with respect to point cloud size suggests that the computation time ratio between the indices remains constant at 40 regardless of the number of points in the point cloud of interest.

## 5 Conclusions

Following the results of the statistical analysis, both holistic indices of structural complexity have strongly correlated values for the forests investigated here. This suggests that ${\mathrm{CE}}^{’}$s underlying theory, namely entropy strongly correlates with ${D_{b}}^{’}$s underlying theory, namely mathematical complexity. Consequently, CE only slightly expands the representation of forest structure compared to the interpretations that can be drawn from $D_{b}$. Until further studies point out that the concept of entropy explains the nature of canopy structure in a sense that mathematical complexity does not, ${\mathrm{CE}}^{’}$s niche cannot be evaluated to be different from ${D_{b}}^{’}$s. Consequently, the next step is to explore if CE has potential to outcompete $D_{b}$ in its niche, offering advantages, that are big enough to outweigh the head start of $D_{b}$.

To replace $D_{b}$ in its niche, CE would have to be more precise, have a wider applicable range, be cheaper or faster in application or offer a new way of interpretability. Precision in detecting forest structure changes is not compared in this study, but both index introductions (Liu et al. 2022; Seidel 2018) cover the precision of their respective index in the sense of the index's ability to distinguish between forest types or tree species. Due to the lack of any reference value of structural complexity, it is not possible to evaluate, if CE is more precise than $D_{b}$.

Because of its linear nature, the main statistical model does not allow for statements about different applicable ranges of the indices. The calculated mixed model on the other hand tends towards suggesting, that ${\mathrm{CE}}^{’}$s upper limit is reached before ${D_{b}}^{’}$s.

CE fails to outcompete $D_{b}$ in speed by a significant margin. Nonetheless, it is important to acknowledge the temporal variability in the significance of computation times. Advancements in technology can render this aspect increasingly obsolete.

The interpretability of $D_{b}$, first through its unit (number of dimensions), secondly through its link to physiology, remains unchallenged. CE yet lacks research that backs the index with $D_{b}$'s level of interpretability, which is possibly explained by the head start of $D_{b}$.

The limitations of this study shed light on the need for future research in this area. Especially, forests of different biomes and climates and forests of structural complexity out of the ranges of this study ($D_{b}>2.4$, $D_{b}<1.5$ or CE>9.5, CE<5, for 15m×15m scans) should be investigated to secure or expand the findings of this study. Also, research into the physiological link of CE to ecosystem functions would significantly expand knowledge about the different underlying theories of both indices.

## Author Contributions

**Tillman Reuter:** conceptualization (equal), formal analysis (equal), investigation (equal), methodology (lead), project administration (equal), software (lead), validation (lead), visualization (lead), writing – original draft (lead), writing – review and editing (equal). **Sebastian Seidel:** data curation (lead), funding acquisition (equal), resources (lead). **Dominik Seidel:** conceptualization (equal), data curation (equal), funding acquisition (equal), project administration (equal), supervision (lead), writing – review and editing (equal).

## Funding

The authors have nothing to report.

## Conflicts of Interest

The authors declare no conflicts of interest.

## Data Availability

The LiDAR point clouds in .laz format as well as the code used to compute the results are available on https://doi.org/10.25625/KQV8C1.
